# Inflammation and Immunophenotypes in Schizophrenia Spectrum Disorders: Current Knowledge and Advances

**DOI:** 10.31083/AP51419

**Published:** 2026-10-23

**Authors:** Julia Karska, Błażej Misiak

**Affiliations:** ^1^Department of Psychiatry, Wroclaw Medical University, 50-367 Wroclaw, Poland

**Keywords:** schizophrenia, psychotic disorders, inflammation, immunophenotyping

Altered immune responses have been investigated as a biological mechanism potentially underlying schizophrenia spectrum disorders (SSDs) for more than 130 years, with SSD research persistently focusing on immunity and inflammation—from the early observations of peripheral leukocyte alterations to contemporary multiomic immunological profiling [1]. In SSDs, inflammation appears to reflect heterogeneous patterns of immunological dysregulation rather than a single uniform inflammatory state, thus playing a role considerably more complex than initially assumed [2]. Current research has begun to delineate specific immune pathways and immunophenotypes that may be particularly relevant for understanding disease mechanisms and guiding future translational efforts. Herein, we summarize immune domains of key importance for SSDs, namely innate immunity, adaptive immunity, and immunometabolic pathways, focusing on their relevance for immunophenotype identification.

Alterations in innate immune pathways are the most extensively investigated aspect of immune dysregulation in SSDs [2]. Recent meta-analytic evidence reveals elevated peripheral neutrophil and monocyte counts in patients with SSDs, especially in first-episode and antipsychotic-naive cases. The fact that these alterations are partially normalized following treatment suggests the early and sustained activation of innate immunity [3]. Neutrophils and monocytes produce proinflammatory cytokines, e.g., interleukin (IL)-1, IL-6, and tumor necrosis factor alpha, which are elevated in patients with both acute and chronic schizophrenia and may contribute to neuroinflammation and symptom exacerbation [3,4,5]. The association of elevated IL-6 levels with a high risk of psychosis suggests that the subclinical activation of inflammatory pathways may precede the onset of overt illness [6]. These findings support the presence of a proinflammatory immunophenotype of SSDs. Functional studies have shown that in patients with SSDs, including decreased cytokine responses of monocytes after stimulation [7] and suppressed functional responses of NK cells [8], which may reflect the functional exhaustion of innate immune responses as the illness progresses and indicate the presence of a distinct immune-exhausted or dysregulated immunophenotype. Furthermore, the dysregulation of innate immune pathways, including the complement system (notably complement component 4 (C4)), toll-like receptors (e.g., toll-like receptor 4 (TLR4)), and altered cytokine signaling, has been associated with mechanisms potentially contributing to SSD pathophysiology. These mechanisms include impaired blood-brain barrier integrity, increased microglial activation, and abnormal synaptic pruning [2,9,10]. However, microglial activation is not always correlated with a high peripheral inflammatory state in SSDs [11,12] and occurs predominantly in the early stages of schizophrenia, when the blood–brain barrier remains intact, and this activation may be modulated by astrocytes [13].

Adaptive immunity in SSDs is mainly characterized by the dysregulation of T cell responses [14]. Although total lymphocyte counts are inconsistently altered across studies, more reproducible evidence highlights dysregulated T helper (Th) cell polarization, which is reflected in elevated interferon gamma, transforming growth factor beta, and IL-17 levels in early-stage psychosis [14]. Subpopulation analyses suggest clinical relevance, with reduced T regulatory cell counts linked to negative symptoms and cognitive impairment and increased Th17 responses associated with more pronounced psychopathology [15,16,17]. Although peripheral B cell counts appear largely unchanged, the presence of autoantibodies targeting synaptic proteins, including N-methyl-D-aspartate receptor, glutamic acid decarboxylase, gamma-aminobutyric acid receptor, neural cell adhesion molecule 1, and neurexin 1, in a subset of patients supports the existence of immune-mediated subtypes within SSDs [18,19]. However, in a large cohort, antibrain autoantibodies in patients with schizophrenia and healthy controls have been identified in similar proportions, with neither their presence nor immunoglobulin subclass independently predicting illness [20]. Adaptive immunity findings further underscore the heterogeneity of immunophenotypes in SSDs, suggesting that these alterations may be associated with distinct inflammatory profiles, depending on their nature and clinical presentation.

In addition to cellular immune mechanisms, the gut–microbiota–brain axis has drawn considerable attention as a potential upstream modulator of immune dysregulation in SSDs [21]. Patients with SSDs exhibit alterations in gut microbial composition accompanied by changes in the production of short-chain fatty acids (SCFAs), including acetate, propionate, and butyrate [22,23,24]. SCFAs are key immunometabolic mediators that regulate intestinal barrier integrity, peripheral immune activation, and microglial function [25]. The reduced availability of anti-inflammatory SCFAs, particularly butyrate, may contribute to the systemic low-grade inflammation, impaired blood–brain barrier function, and aberrant neuroimmune signaling observed in SSDs [25]. Although current findings remain preliminary, the microbiota–SCFA pathway represents a promising mechanistic link between environmental exposure, immune heterogeneity, and neurodevelopmental vulnerability in psychosis, potentially contributing to immunophenotypic variability. These findings warrant further longitudinal and interventional research.

The concept of immunophenotyping in SSDs is supported by the fact that patients can be stratified into subgroups with high versus low levels of certain peripheral inflammatory markers [26,27]. Functional studies demonstrating reduced immune-cell responsiveness to stimulation further suggest that inflammation in SSDs may be paradoxical, i.e., simultaneously heightened in certain aspects and suppressed in others [7,8]. As mentioned earlier, this heterogeneity may reflect a form of immune burnout or functional exhaustion within the immune system. Early-life stressors, including infections or childhood adversity, may induce genetic and epigenetic modifications that, over time, accelerate processes resembling physiological immunosenescence [14]. This state would be characterized by alterations similar to those observed in natural immunosenescence, particularly with respect to dysregulated inflammatory responses and elevated cytokine levels [14]. In the context of innate immunity, these changes may also be interpreted as the dysregulation of trained immunity, whereby the memory-like responses of innate immune cells become maladaptively reprogrammed [28]. Although no direct evidence demonstrating an overlap between immunosenescence and the development of SSDs is available, the similarities between these processes suggest that an immunosenescence-like mechanism may contribute to the emergence of psychosis [14]. However, more detailed and longitudinal studies are required to validate this theory.

Despite substantial progress in characterizing immune alterations in SSDs, several challenges remain. A key priority is the transition from group-level inflammatory findings toward clinically actionable immunophenotyping frameworks capturing the marked heterogeneity of immune profiles in SSDs. Two directions are particularly relevant, namely longitudinal studies integrating immune markers with clinical trajectories to identify stable immunophenotypes and stratified interventional studies targeting specific immune pathways in biologically defined subgroups. Given the greater plasticity of immune alterations in the early phases of psychosis, these phases may be especially informative. Ultimately, immunophenotyping may support patient stratification and targeted therapeutic development, helping move the field beyond descriptive inflammation research toward meaningful translational impact.
